# Methods for broad‐scale plant phenology assessments using citizen scientists’ photographs

**DOI:** 10.1002/aps3.11315

**Published:** 2020-01-08

**Authors:** Vijay V. Barve, Laura Brenskelle, Daijiang Li, Brian J. Stucky, Narayani V. Barve, Maggie M. Hantak, Bryan S. McLean, Daniel J. Paluh, Jessica A. Oswald, Michael W. Belitz, Ryan A. Folk, Robert P. Guralnick

**Affiliations:** ^1^ Florida Museum of Natural History University of Florida Gainesville Florida 32611 USA; ^2^ Department of Biology University of North Carolina Greensboro Greensboro North Carolina 27402 USA; ^3^ Biology Department University of Nevada Reno Nevada 89557 USA; ^4^ Department of Biological Sciences Mississippi State University Mississippi State Mississippi 39762 USA

**Keywords:** anomalous flowering, citizen science, data integration, iNaturalist, plant phenology, *Yucca*

## Abstract

**Premise:**

Citizen science platforms for sharing photographed digital vouchers, such as iNaturalist, are a promising source of phenology data, but methods and best practices for use have not been developed. Here we introduce methods using *Yucca* flowering phenology as a case study, because drivers of *Yucca* phenology are not well understood despite the need to synchronize flowering with obligate pollinators. There is also evidence of recent anomalous winter flowering events, but with unknown spatiotemporal extents.

**Methods:**

We collaboratively developed a rigorous, consensus‐based approach for annotating and sharing whole plant and flower presence data from iNaturalist and applied it to *Yucca* records. We compared spatiotemporal flowering coverage from our annotations with other broad‐scale monitoring networks (e.g., the National Phenology Network) in order to determine the unique value of photograph‐based citizen science resources.

**Results:**

Annotations from iNaturalist were uniquely able to delineate extents of unusual flowering events in *Yucca*. These events, which occurred in two different regions of the Desert Southwest, did not appear to disrupt the typical‐period flowering.

**Discussion:**

Our work demonstrates that best practice approaches to scoring iNaturalist records provide fine‐scale delimitation of phenological events. This approach can be applied to other plant groups to better understand how phenology responds to changing climate.

Plant phenology—the timing of plant life cycle stages such as flowering or leaf senescence—plays a critical role in terrestrial ecosystems and is known to be responsive to environmental changes (Rathcke and Lacey, 1985; Ollerton and Lack, 1992; Cleland et al., 2007; Chuine, 2010). The fingerprint of accelerating global change, including both global‐scale climatic changes and their local‐scale outcomes, along with human disturbance, may show its first biotic signs in disrupted phenologies. These disruptions can have significant consequences if they lead to phenological mismatches between plants and the animals that depend on them (Kudo and Ida, 2013; Mayor et al., 2017).

Plant phenology data that cover broad scales have until recently only been available through monitoring networks, such as the National Phenology Network (NPN) (Schwartz et al., 2012; Rosemartin et al., 2014) in the United States, which coordinates amateur and professional scientists to make phenological observations. Although such networks provide critical data, reporting remains sparse because such networks often focus on key taxa or repeat sampling at a relatively small number of locations. Promising new resources, as well as historical resources (e.g., imaged herbarium sheets), that provide wider taxonomic and spatial coverage are becoming available digitally (Davis et al., 2015; Willis et al., 2017; Silva et al., 2018); however, these have attendant issues with sampling protocols and with proper annotation of phenological traits and species identification.

An alternative set of resources that has yet to be broadly tapped for phenology studies comprises repositories of naturalist citizen science images. Here we focus in particular on iNaturalist (http://www.inaturalist.org/) as a source of phenology data because it: (1) enforces the provision of species occurrence metadata required for scientific use; (2) manages taxonomic resources, putting a premium on quality identification, and sets objective requirements for records to be considered “research grade”; (3) allows reporting of cultivation status along with annotation of traits including phenology in metadata fields, although trait annotation is still not often used; (4) is growing at a rapid and increasing pace in terms of records and species represented; (5) is directly connected with other global species occurrences aggregators such as the Global Biodiversity Information Facility (GBIF), thus ensuring longer‐term integration and sustainability. Despite these promising attributes, best practices for use of iNaturalist and associated citizen science data resources must still be developed to realize the full value of these data streams for assessing plant phenology trends.

Here we provide a particularly salient case study, focusing on the plant genus *Yucca* L., a perennial shrub or tree with distinctive flowers. *Yucca* has highest diversity in arid southwestern North America, but is broadly distributed over most of continental North America. We focus on *Yucca* for three reasons. First, *Yucca* are commonly photographed and provide an excellent test case for developing proper practices for reporting needed information on phenology state. Second, *Yucca* have highly specific, co‐evolved obligate pollinators and herbivores, the *Yucca* moths in the family Prodoxidae, and thus, their phenologies must synchronize to their pollinator in order to set fruit (Rafferty et al., 2015). However, the proximal environmental cues that determine *Yucca* phenology have only been examined for a few species and locations in the Desert Southwest, with evidence from those studies pointing to climatic factors as determinants of the timing of inflorescence (Smith and Ludwig, 1976; Ackerman et al., 1980). Third, recent reports of anomalous flowering events in *Yucca* (Cornet, 2019) during fall and winter, and well outside of typical flowering periods (MacKay, 2013), raise the possibility that climatic changes may impact fitness, because yucca moth pollinators are presumably not yet on wing during these events. One such event was reported for Joshua trees (*Yucca brevifolia* Engelm.) in Joshua Tree National Park, but it is unknown if the spatial, temporal, and taxonomic extent was broader than these initial reports. Finally, it is unknown if those populations displaying anomalous events also experienced disruption of the typical flowering phenology periods.

The first aim of our study was to derive generalized best practices for gathering phenological information from the citizen science records available from iNaturalist. As a test case, we used these general guidelines to develop a genus‐specific phenology scoring rubric for *Yucca*. With this rubric, we (1) record phenological data from nearly 9000 images of *Yucca* from iNaturalist, (2) compare these data with those available from the NPN and National Ecological Observatory Network (NEON; Elmendorf et al., 2016) in terms of spatial coverage and utility, and (3) provide an informatics workflow for integration of annotated specimens using the Plant Phenology Ontology and its associated data portal (i.e., the Global Plant Phenology Data Portal; https://www.plantphenology.org). Our second aim is to reconstruct *Yucca* phenology patterns to determine: (1) which species flowered at anomalous times (e.g., in autumn, well outside the typical spring or summer bloom period), and (2) the spatial and temporal extents of those anomalies and the possible impact on more typical flowering periods. We further provide a framework for downstream testing of climate factors that might determine timing of inflorescence development (i.e., flowering) in *Yucca* that can be used in future phenological investigations.

## METHODS

### Data accumulation

Initially, all iNaturalist records available for the genus *Yucca* through 15 February 2019 were downloaded using function download_images from imageNat (Barve, 2019; R Core Team, 2019), a newly developed R package that was built for this effort. There were approximately 23,600 of these records, 17,500 of which were “research grade.” Research‐grade records are verifiable observations where at least two participants agree about taxon identity. Verifiable observations are observations that are georeferenced, have a date of observation listed, include photos or sounds, and are not recorded as captured or planted. After exploring the number of records available per species, we decided that only species with at least 100 research‐grade records would be considered further, given our interest in ultimately developing models to predict timing of flowering; 14 species met this criterion. We also decided that species with >1000 research‐grade records available did not need to be exhaustively sampled for the purposes of this work, and instead we randomly selected for scoring 1000 records per species from the full set of records. All images were downloaded at the highest possible resolution available from iNaturalist in order to assure effective detection of phenology traits.

After the preliminary analysis of phenology data across 14 *Yucca* species, additional data for three species (*Y. baccata* Torr., *Y. brevifolia*,* Y. schidigera* Ortgies) were downloaded for the period of February to May 2019. These records cover the majority of the time period considered to be within the typical flowering period for these species and provided a means to examine spatial patterns of flowering phenology in the spring of 2019, after atypical flowering periods the previous fall and winter. We restricted our taxon set for further analyses presented here to six *Yucca* species (shown in Table 1), five of which are also monitored by NPN or NEON, to facilitate direct comparisons with those data resources. We also included similar, detailed analyses for one other taxon, *Y. filamentosa* L., which is not monitored by other resources, but which was particularly illustrative of the challenges both in implementing phenology scoring best practices and in data quality issues with iNaturalist records.

**Table 1:** Typical flowering period for the six focal species covered with overlaps between iNaturalist and the National Phenology Network.

Species name	Common name	Flowering period	Sources
*Yucca baccata*	Banana yucca	March–July	1, 2
*Yucca brevifolia*	Joshua tree	March–May	1
*Yucca elata*	Soaptree yucca	May–July	1
*Yucca filamentosa*	Common yucca	April–August	2
*Yucca glauca*	Great Plains yucca	May–July	1
*Yucca schidigera*	Mojave yucca	March–May	1

^1^ https://www.feis-crs.org/feis/, with searches for specific species and collation from seasonal development descriptions.

^2^ https://www.wildflower.org/plants/, with searches for specific species and collation from “bloom information” field.

### Developing best practices for gathering phenological data from iNaturalist photographs

Two authors (V.V.B. and R.P.G.) initially examined the *Yucca* photographs from iNaturalist and developed a rubric for scoring these images, focusing on flower and whole plant presence (Appendix 1). Definition of traits for this rubric was based on work from Brenskelle et al. (2019) and Stucky et al. (2018), who have developed a set of formal definitions in the Plant Phenology Ontology. In particular, we scored flowers, open flowers, and whole plants as present or absent. We added “uncertain” as a scoring category, because initial examination revealed cases where image quality was poor or the state otherwise difficult to observe.

We recruited nine additional graduate students or postdoctoral fellows (besides V.V.B. and R.P.G.) as part of a semester‐long project at the Florida Museum of Natural History (FLMNH), most of whom were not familiar with *Yucca* before beginning the project. After a first presentation of the scoring rubric, 11 scorers were given the same 100 iNaturalist images to score as a test for consistency. We reconvened to discuss photographs where among‐scorer conflict existed and reached a group consensus of how these cases were to be scored. After a series of refinements based on inter‐scorer comparisons, the 11 scorers were each given a new, identical set of 100 more records to test consistency again. At this point, scoring was greater than 90% consistent among scorers across species and traits. Finally, after these practice sets, each scorer was assigned two or three sets of 1000 images to score on their own. The end result is that each image was scored by three independent volunteers, which is the minimum number required to both determine any potential scorer conflict and generate a majority rule assessment.

Initial scoring work was done on spreadsheets, but to hasten and help automate steps in the scoring process, one of us (B.J.S.) developed a software tool called ImageAnt (https://gitlab.com/stuckyb/imageant) to help with the task, which will be described more formally in a separate contribution. ImageAnt uses a simple language so that users can define transcription targets and an order of scoring. To help make image annotation as efficient as possible, ImageAnt can use the answers to high‐level questions to decide which lower‐level questions to display. For example, if the user indicates that flowers are present on image, ImageAnt will not ask whether the photograph is of a whole or portion of a plant. Whether a photograph shows a whole plant or portion of a plant is only relevant in cases where flowers are absent. The ImageAnt software then saves annotations as a CSV file.

During the scoring effort, we continued to identify difficult cases and addressed those issues through iterative refinements. In particular, for the full scoring effort, we added a new scoring category in ImageAnt that enabled scorers to flag records they found particularly problematic, allowing us to go back and review these records as a group. These flagged records were different from those recorded as “uncertain”; in many cases, annotators were certain in their uncertainty.

Some important decisions were made prior to the classification process that affected how images were scored and future applications of the data set. For example, we decided not to choose a single focal plant in an image because, in some cases, there are other plants *of the same species* in the background but with a different phenological stage. To be clear, we only scored the target species (that is, the species in the photograph matching species metadata) and did not consider other *Yucca* species even if co‐occurring in the same digital photograph voucher. If at least one whole plant of the target species was in view in an image, the image was scored as “whole plant present.” Similarly, we scored flowers and open flowers present if any plant of the target species (whole or partial) in the image had those states present. Appendix S1 provides full details on scoring practices.

In sum, three independent scores were captured for each of the 8575 total images. After a further vetting to remove a subset of images that could not be scored (discussed in Results), we compiled a scored annotation data set for each photograph. Next, in cases of conflicting reports of presence or absence of a trait, a smaller group (V.V.B., R.P.G., and L.B.) individually reviewed those inconsistencies case‐by‐case. After that review, the smaller group reached a consensus decision among themselves, and finalized a score for those records. V.V.B., R.P.G., and L.B. also individually scored phenology for a spring 2019 subset of records (discussed above), and then collectively reconciled results among themselves to derive a final vetted annotation. Finally, we abstracted a set of general best practices (Appendix 1) usable for any phenology scoring project.

### Comparing iNaturalist, NEON, and NPN phenologies and identifying phenological anomalies

**Figure 1:**
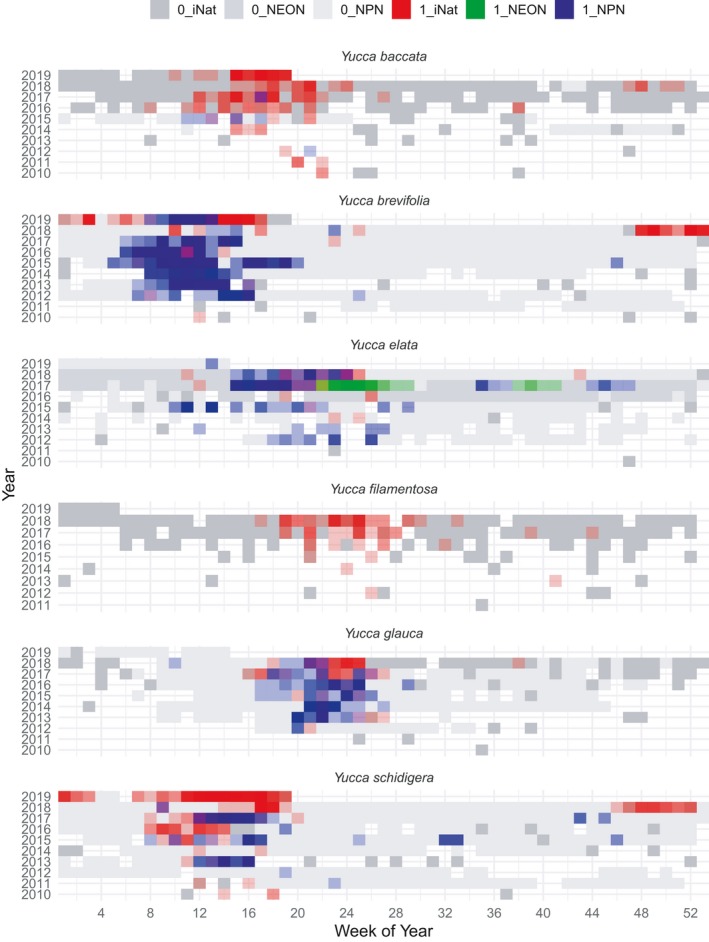
Flowering of the six focal *Yucca* species is shown with colored boxes for each week of the year from 2010–2019. Flowering absences are shown in different intensities of the color gray for different data resources (darkest for iNaturalist, lightest for National Phenology Network [NPN]). Flowering presences are indicated with different colors to identify whether these were documented by iNaturalist (red), NEON (light green), or NPN (blue). The coloring intensity indicates the number of reports from a given source during a specific time period. The colors are mixed when there are flowering presences reported from multiple sources for a single week.

**Figure 2:**
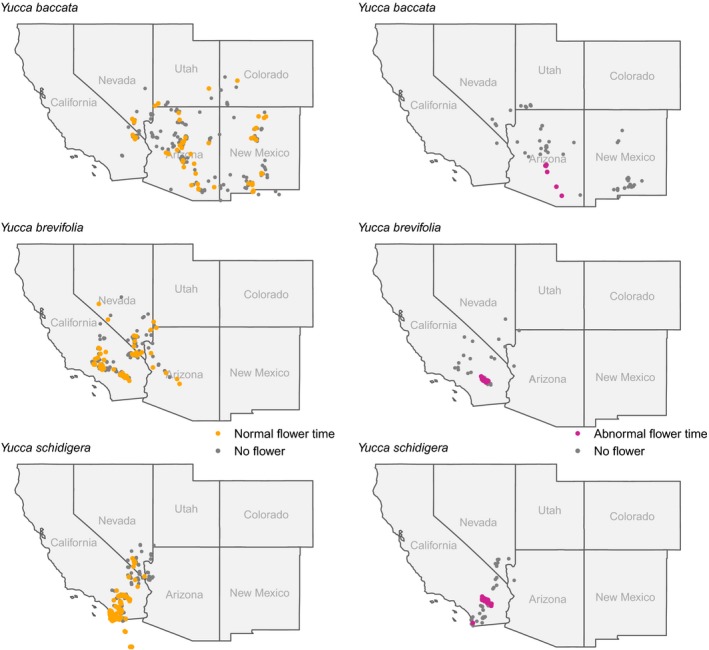
The spatial distribution of flowering of three yucca species in fall–winter 2018 and spring 2019. Typical flowering time (yellow dots) in spring 2019 is defined broadly here to capture potential early onsets, and ranges from 11 February to 15 May 2019. We delineate the anomalous flowering times (magenta dots) as those occurring from 1 November 2018 to 10 February 2019, well outside typical known time frames. These are superimposed on non‐flowering records (gray dots) from the same time periods. Points outside of the map boundary (e.g., for *Y. schidigera*) are located in Mexico, which is not shown here.

We downloaded phenology observation data from the NPN (https://www.usanpn.org/usa-national-phenology-network) and NEON (http://www.neonscience.org) for all *Yucca* species and from the Global Plant Phenology Data Portal (https://www.plantphenology.org), which provides a harmonized set of reporting. Table 2 provides a summary of the number of observations of whole plants, or annotations from photographed plants, for each source. We also plotted and compared spatial distributions of phenology reporting for all sources, along with temporal trends in data sets from 2014–2019 for overlapping species (Figs. 1, 2). In particular, we examined spatial and temporal patterns of anomalous blooms in fall and winter of 2018–2019 for those species with data from multiple phenology observation resources. We defined anomalous flowering as flowering well outside known bloom periods; Table 1 provides a summary of typical flowering period, along with citation source, for the key six species (noted above) with overlapping data in all repositories. Any flowering found well outside of those ranges of time was considered anomalous. As a clear example, there were multiple sightings of *Y. brevifolia* in flower in November 2018, which is well outside its typical bloom period in March–May. In order to visualize whether those areas with anomalous bloom periods also had *Yucca* flowering events during the typical blooming period, we plotted spatial patterns of blooming constrained by different time periods (Fig. 2).

**Table 2:** Number of data points for each focal species that had one or more sources.

Species name	iNaturalist	NPN	NEON
*Yucca baccata*	902	72	0
*Yucca brevifolia*	1213	17,367	0
*Yucca elata*	2845	3730	3499
*Yucca filamentosa*	417	0	0
*Yucca glauca*	384	15,478	0
*Yucca schidigera*	1222	3533	0

Note

NEON = National Ecological Observatory Network; NPN = National Phenology Network.

### Publishing iNaturalist phenology annotations

In order to make our *Yucca* phenology scores available in a reusable format, they were uploaded to the Global Plant Phenology Data Portal. In preparation for ingestion, these data were converted into a spreadsheet with associated iNaturalist's uniform resource identifiers (URI), observation metadata such as the location and date of the observation, and descriptions of phenology using terminology from the Plant Phenology Ontology (Stucky et al., 2018). In this first round of provisioning data to the Global Plant Phenology Data Portal, we did not provide scoring reconciliation metadata. That is, we did not provide metadata indicating scoring conflict in records or how these were resolved. However, such metadata are important to report; in future iterations we plan to provide information about the scoring process. Once the data were reformatted for ingestion, they were uploaded to the portal using the ingestion pipeline developed for the Plant Phenology Ontology, described further in its GitHub repository (https://github.com/biocodellc/ppo-data-pipeline).

## RESULTS

### Developing a scoring rubric and outcome of *Yucca* scoring

We developed a general approach to scoring iNaturalist photographs, which is summarized in Appendix 1. This approach includes a set of best practices that we recommend be followed in order to create research‐quality phenology data from online photographic resources. We implemented this approach, leveraging effort from 11 trained volunteers (all included as authors) who each scored between 2000 and 3000 images for all three traits of interest (i.e., whole plant presence, flower presence, and open flower presence). All 8575 photographs were annotated by at least two people, and most were scored by three. After initial scoring, a subset of photographs that could not be scored at all were removed, leaving a total of 8129 photographs that were assembled into a final data set that captured phenology reporting for 14 *Yucca* species. We focus hereafter on the six species that overlap with reporting from the NPN.

**Figure 3:**
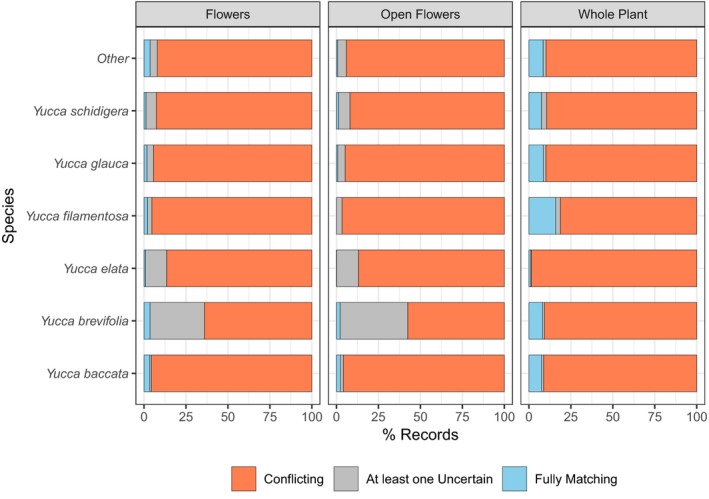
Percentage of cases of full matching, conflicting, and uncertain records per species. Uncertain cases are those in which at least one classifier reported they could not ascertain presence or absence. Larger, tree‐form *Yucca* species often have increased rates of uncertainty in documenting flowers, whereas some smaller shrub species (e.g., *Y. filamentosa*) proved challenging for documenting whole versus portion of a plant.

Figure 3 shows results for scorer consistency, both per species and overall, after initial practice rounds and using the fully developed rubric. We expected that whole plant assessment would be easier for *Yucca* species that are more branched and tree‐like, such as *Y. brevifolia* and *Y. elata* (Engelm.) Engelm. However, those larger species are sometimes photographed from a farther distance, making it challenging to assess flowering phenology. Overall, our results show that scores of flowering phenology as “uncertain” were indeed highest in the largest species, *Y. brevifolia* (30–40%), and the second largest species, *Y. elata*, also had moderately frequent uncertain scores (10%). As expected, we found the greatest conflict among scorers in detecting whole plants versus partial plants, as this can be challenging in photographs where the ground is not visible due to dense undergrowth or the angle of the photograph, or where clonality makes it unclear whether a whole individual was captured, all of which happened frequently. *Yucca filamentosa* was the most challenging to score as whole versus partial plant based on its unusually high inter‐scorer conflict (>15%), likely because it is located in denser, more mesic habitats in the southeastern United States with greater plant crowding. Despite species variance, the overall rate of uncertain scoring was still relatively low, below 1–2% in a majority of species. Inter‐scorer conflict was also relatively low for flowering traits, which provides evidence that the scoring rubric could be used successfully in most cases, and that the primary conflict was in assessing whether a complete individual was captured rather than recording the phenology states in the photograph.

Table 3 shows per‐species proportions of whole plants recorded for those photographs where flowers are absent. We were particularly interested in whether photographers generally try to take photos of whole plants, as these photos are most useful for demonstrating absence of flowers. Indeed, more than nine out of ten photos are of whole plants, with no discernable bias for more or less whole plant reporting in larger, branched species and smaller, stemless shrub species. We also expected a relatively high percentage of photographs with plants in flower, given known observation bias toward recording flowering individuals (Panchen et al., 2019). Excluding *Y. elata*, we found that the percentage of the target *Yucca* species in flower on iNaturalist ranged from 16–26%. In the case of *Y. elata*, one iNaturalist photographer was extremely active in photographing that species whether in bloom or not, resulting in a much higher rate of recorded flower absence. Taken together, our results suggest a general bias toward observers photographing plants in flower.

**Table 3:** Proportion of whole plant photographs for those records with flowers absent and overall proportion of photographs with flowers (whether opened or unopened).^a^

Species	Proportion of whole plant photographs	Proportion of flower photographs
*Yucca baccata*	0.93	0.20
*Yucca brevifolia*	0.90	0.23
*Yucca elata*	0.98	0.06
*Yucca filamentosa*	0.93	0.17
*Yucca glauca*	0.95	0.16
*Yucca schidigera*	0.90	0.26

a Most photographers are capturing whole plants, and are biased toward those plants with flowers, as discussed in the text.

### Comparison of different observation network reporting of *Yucca* phenology

**Figure 4:**
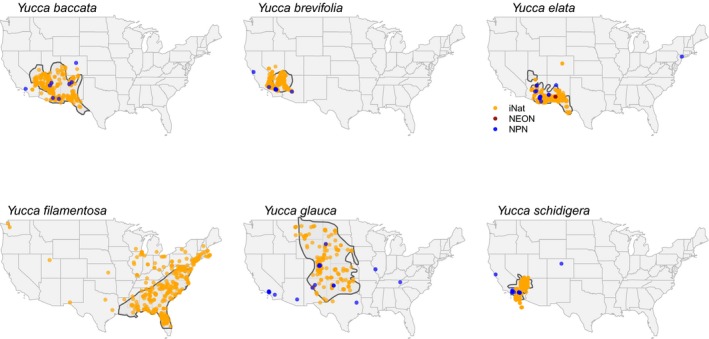
The spatial distribution of occurrences of the six focal *Yucca* species shown with different colored dots indicating the occurrence source. Species’ range maps from eFlora.org were digitized and included in the background.

Figure 4 shows the spatial coverage of records from different phenology sources. Two patterns are immediately visible. First, iNaturalist records provide significantly more spatial coverage of phenology than NPN or NEON. That spatial coverage comes at the expense of repeat temporal coverage that is lacking in iNaturalist and a critical strength of NPN. For each NPN site, there are often hundreds of repeat measurements of the same individual or populations. However, NPN also includes many repeat‐sampled populations that are outside the native ranges of the species sampled, likely representing either cultivated specimens or misidentifications. This is most clear for *Y. schidigera* and *Y. glauca* Nutt. In *Y. schidigera*, the majority of sites where observations occur are in the core part of the range, but there are two sites, one in northern Colorado and one in the Bay Area of California, that are well outside known distribution areas and must be cultivated specimens. In *Y. glauca*, the Great Plains yucca, multiple reports in Southern California as well as Tennessee and south‐central Arizona are all well outside the known range of the species (Althoff, 2016). Unfortunately, reporting about whether these are cultivated or wild populations cannot be easily determined in the NPN data sets.

### Documenting anomalous flowering in space and time

*Yucca brevifolia* and *Y. schidigera* both show a strong signal of blooming in mid‐November 2018 that continued through January 2019 (Fig. 1), which is well outside typical bloom timing (Table 1). Anomalous flowering is also seen in *Y*. *baccata*, the banana yucca, for a shorter duration during November and December 2018. When the anomalous bloom is mapped spatially, it is striking that anomalous blooming events were spatially restricted in all three species. For *Y. brevifolia* and *Y. schidigera*, the anomalous flowering is restricted to areas in and around Joshua Tree National Park, with the exception of two *Y. schidigera* with flower buds observed in very late January in San Diego County (Fig. 4). *Yucca baccata*, the range of which does not overlap with the previous two species, also experienced anomalous blooming events during fall–winter 2018 in south‐central Arizona, well outside the typical period of March–July.

### Publishing scoring results to the Global Plant Phenology Data Portal

In order to make iNaturalist‐derived phenology annotations openly available, the Global Plant Phenology Data Portal was reconfigured to add iNaturalist as a source for phenology annotations. We labeled the source as “Image Scoring Records from iNaturalist,” and results returned are provided as either a map or table, all available for download from the Global Plant Phenology Data Portal. The records are also available via the R package “rppo.” In all cases, the individual record results always point back to a URL for the observation record in iNaturalist, including the photograph from which the annotation was made. Appendix S2 provides a screenshot showing an example of a result return for all 14 scored *Yucca* species with open flowers present, showing mapped results.

## DISCUSSION

### The need for phenology scoring best practices from citizen science photographs

We provide a brief best practices summary for developing plant phenology scoring methods for online, citizen science photography sharing platforms in Appendix 1. We also provide a *Yucca*‐specific rubric in Appendix S1. As Appendix 1 discusses in more detail, key practices include aligning scoring to well‐defined terms in ontologies, developing an iterative scoring process, working collaboratively with volunteer scorers, and using annotation tools to increase efficiency. These best practices are essential, because scoring whole versus portions of plants and flowering traits proved surprisingly challenging for *Yucca*, based on not only our quantitative results of inter‐scorer comparisons, but also the sometimes energetic discussions that arose. It is also critical to allow uncertainty to be reported, especially in tree‐like species, where photographing the whole plant often resulted in reduced resolution and associated difficulty identifying flowering stage. Finally, we note that iNaturalist itself has reporting mechanisms for plant phenology, so a longer‐term goal is to assure that scoring efforts work in both directions, such that annotations can ultimately be fed back to iNaturalist, and to encourage further effort to denote phenology by the more than 500,000 citizen scientists who contribute to that platform.

We doubt that the scoring challenges discussed above are unique to *Yucca*, necessitating that best practices should be defined broadly. Additionally, developing a more detailed guide to *Yucca* scoring (Appendix S1) provided needed specificity for taxon‐specific scoring challenges. As an example, our iterative, consensus‐based approach led to recognition that scoring of whole versus a portion of a plant was not independent of flowering scoring. For example, it was necessary to determine whether or not the flowering stalk was living or dead in order to score whole plant presence. If the stalk was dead, but not completely in view, we scored whole plant present. Conversely, if the stalk was alive but not fully visible, we scored whole plant absent. We also quickly determined that scoring whole versus part of plant cannot be done independent of species‐level taxonomic knowledge because expected differences in growth form (e.g., clonality, caulescent habit) are needed to assess the presence of an entire individual. This proved especially important in cases where it is difficult to determine the plant's position relative to the ground, which can happen in photographs with multiple species or of landscapes. Marginally less difficult was scoring buds versus open flowers. For that rubric, we only scored unopened flowers present in cases where a bud was clearly visible. In many cases, a stalk had formed and bracts were visible but not buds; these were scored as flowers absent. Transitional states between unopened and opened flowers and between opened flowers and senesced flowers (here scored as flowers absent) are always challenging, and more details about our rubric for those states are provided in Appendix S1.

### The unique value of iNaturalist for providing spatial flowering phenology coverage

A guiding question that motivated our research was whether the coverage of iNaturalist could improve understanding of flowering phenology pattern and process compared to what is available from NPN and NEON, which are the key monitoring data sets usable for generating phenoclimatic models, especially in the United States. Previous work examining flowering phenology in *Yucca* has been limited to just a few sites and years (Smith and Ludwig, 1976; Ackerman et al., 1980), and no explicit models have been developed to determine if climate factors, photoperiod, or the interaction between the two can be predictive of flowering time. This question is particularly important, because if climatic factors do control *Yucca* flowering phenology timing, it is possible that climate change could create mismatches between *Yucca* and their obligate moth pollinators (Rafferty et al., 2015).

A key finding when comparing iNaturalist records to NPN and NEON monitoring is the much broader spatial extent of records found in iNaturalist. iNaturalist records may provide a good example of the power of observations collected over a gradient, as opposed to those collected via repeat sampling. Detecting non‐linear trends may be greatly improved with such broad‐scale sampling, as opposed to more replication across fewer sites (Kreyling et al., 2018). Additionally, we found that iNaturalist records are almost all within the core known range of species, which is not the case with the more sparsely spatially sampled NPN records. Finally, NPN records for *Yucca* sometimes appear to be cultivated specimens, but limited reporting makes detection of these cultivated specimens challenging.

Similar issues with cultivated specimens also occur on iNaturalist, but it is simple to tag records as cultivated, and those listed as such cannot become “research grade.” Although we have not undertaken a quantification of how many clearly cultivated or planted specimens are not labeled as such and become research grade, a cursory examination of research‐grade *Yucca* observations suggests that the vast majority are indeed non‐cultivated. Furthermore, because spatial sampling in iNaturalist is much larger than temporal sampling, the problem with cultivated specimens in relation to phenology patterns may be less acute. For example, the NPN reports an unusual flowering period for a specimen or specimens of *Y. elata* in the Joseph Wood Krutch Garden on the University of Arizona campus in the fall of 2017. This site had multiple reported days of flowering over two periods, in late August/early September, and again over multiple days in November, and is clearly visible on the *Y. elata* plot in Fig. 1. No other sites for *Y. elata* recorded this unusual bloom pattern. Cultivated specimens, especially in managed gardens such as the one on the University of Arizona campus, may experience different conditions (e.g., watering regimes) from non‐managed plants, desynchronizing normal phenology processes (Buyantuye and Wu, 2012). Unless information about those management regimes is known and can be included in models, such records may ultimately obscure understanding of drivers of flowering phenology. Differentiation of natural flowering anomalies from those occurring due to cultivation is both critical and challenging, especially in cases such as data from NPN, given lack of reporting methods and photographs usable for judging surrounding landscape.

### Documentation of restricted anomalous flowering in *Yucca*

Our work shows clear evidence of anomalous flowering in fall 2018 for three *Yucca* species. In all cases, that anomalous flowering is spatially restricted in extent, occurring in Joshua Tree National Park for *Y. brevifolia* and *Y. schidigera*, and in south‐central Arizona for *Y. baccata*. In the case of anomalous flowering in and around Joshua Tree National Park, the only two *Yucca* species found there are both affected. However, *Y. baccata* is sympatric with other *Yucca* species, including *Y. elata*, which apparently did not show this anomaly (or it was not sampled). While it remains possible that sampling deficiencies have limited detection of the spatiotemporal extent of anomalous blooms, absences are well documented across the range in other areas over both the anomalous and typical flowering period, and no other years show strong evidence of concerted anomalies as seen in the fall and winter 2018.

A key question is the cause of anomalous blooms, and it has been speculated that an anomalously colder and wetter fall in 2018 across portions of the Desert Southwest of the United States may have triggered this event (Moore, 2018), but this has yet to be tested rigorously. Climate factors have been previously hypothesized to affect phenology of *Yucca*. Smith and Ludwig (1976), for example, found that *Y. elata* populations at a site near the U.S. Department of Agriculture Jornada Experimental Range in southern New Mexico formed stalks almost a month later in 1973 compared to 1972, and speculated that this delay was caused by a wetter, cooler spring. However, Ackerman et al. (1980) claimed that in *Y. schidigera*, flowering may be driven more by photoperiod. Our results cast doubt on a purely photoperiod‐driven phenological response, given differences in photoperiod in December when desert *Yucca* species were found in bloom in 2018, versus typical bloom timing in March and April. Both suggest the importance of other proximal climatic drivers. However, a more thorough test of such climate drivers requires a much more thorough examination of overall phenology across well‐studied *Yucca* species and multiple years of data. Particular attention to localized areas of high rainfall and unusual cold, especially in relation to spatially restricted anomalous flowering, is especially warranted.

A final question we address here is whether anomalous *Yucca* blooming meant that areas where those blooms occurred had normal flowering patterns in the typical blooming period. If so, then such anomalies may have strong fitness consequences, especially because flower production requires considerable energy output in *Yucca*, and often these plants cannot flower each year due to trade‐offs between optimizing vegetative versus floral growth (Smith and Ludwig, 1976). Figure 4 provides clear evidence that those areas with anomalous blooms also had plants flowering at typical times. Although it is unlikely that anomalous flowers are pollinated given the presumed absence of its pollinator, this too requires a more thorough examination to verify. It may be that climatic cues are synchronized between *Yucca* and their obligate pollinator moth and that the unusual flowering timing is adaptive, allowing yuccas to take advantage of the right conditions for pollination. However, Rafferty et al. (2015) suggest that mutualisms between *Yucca* and their pollinating moths are not necessarily synchronized to climate cues, at least in typical spring flowering conditions. Further examination of whether any plants that were photographed formed fruits during the period between unusual and usual flowering would help provide evidence for the intriguing question of whether adult pollinators were also present.

### Caveats and conclusions

Our work demonstrates that iNaturalist records provide a useful resource for phenology studies, if these are scored carefully following a well‐designed rubric. Here we have focused on the ability of iNaturalist records to uncover spatial and temporal trends in flowering, especially the ability to localize where and when anomalous *Yucca* flowering occurred in fall and winter 2018 after reports of such events in the media. Although our results strongly show the value of iNaturalist data in answering such spatiotemporal phenology questions, we close with a few needed caveats regarding use of these data. First, while identifications are generally good for the focal taxa used in this study, despite the general challenge of field identification of *Yucca* (McKelvey and Sax, 1933), there are still cases where research‐grade specimens are misidentified. Although evaluating identifications was not explicitly part of our efforts, and none of the participants in this study are experts in taxonomic identification of *Yucca* from photographs, we noted a very small percentage of obvious misidentification for the six focal species (well below 1%). This low rate likely reflects in part our choice of particularly easily identified taxa such as *Y*. *brevifolia*. However, as the *Y. filamentosa* panel in Fig. 4 shows, there remain spatial outliers in iNaturalist that fall outside the known species range as documented by Flora of North America (1993), which likely indicate cultivated observations (discussed above) that are not labeled as such, or potentially misidentifications. Finally, while extremely rare as most uploads come from cameras with automated date stamps, we did find cases (e.g., upload dates preceded dates of the photograph being taken) where camera date and times may have been improperly set or dates were improperly entered manually.

Like all occurrence data sets reused from aggregators, care must be taken in order to flag problem records and outlier information. Our efforts at vetting data and making our results immediately available on the Global Plant Phenology Data Portal provide not only a mechanism for re‐use but also a means to assure that data can ultimately be improved. Finally, publishing iNaturalist records to the Global Plant Phenology Data Portal also extends data integration mechanisms across multiple types of data sources, which now include phenology observations from monitoring networks such as NPN, phenology annotations from herbarium specimens, and now citizen science–based photographic evidence from the iNaturalist platform. We also note the potential for such data sets to serve as inputs into rapidly developing supervised machine learning approaches (Lorieul et al., 2019) to scale up phenology reporting in the future.

## AUTHOR CONTRIBUTIONS

All authors conceived the idea together. V.V.B. performed data collection for this study. V.V.B. and R.P.G. did the initial examination of photographs to check feasibility. Initial scoring was done by V.V.B., L.B, D.L., N.V.B., M.M.H., B.S.M., D.J.P., J.A.O., R.A.F., M.W.B., and R.P.G. V.V.B., L.B., and R.P.G. reconciled the scoring. B.J.S. developed the scoring tool. Data analysis was done by V.V.B and D.L. Final data was generated from the Scores file by L.B., V.V.B., and R.P.G., and was then uploaded to the National Phenology Network. R.P.G. led the writing of the manuscript with V.V.B., and all authors helped with editing.

## Supporting information


**APPENDIX S1.** A scoring guide for documenting *Yucca* phenological traits examined here, including whole plant presence and flower presence.

**APPENDIX S2.** A screenshot showing additions to the Global Plant Phenology Data Portal based on our work. Phenology annotations created by this project are discoverable by choosing the “Image Scoring Records from iNaturalist” source and searching for the genus *Yucca*. The portal returns a map interface by default, but records can be viewed in table‐mode and downloaded.

## Data Availability

The finalized *Yucca* data are available on the Global Plant Phenology Data Portal (https://www.plantphenology.org) and can be searched there. Data integration conversion scripts used to convert *Yucca* scoring to finalized data on the portal are on Github (https://github.com/biocodellc/ppo-data-pipeline/tree/master/projects/image_scoring). Finally, we have compiled a citable version of iNaturalist data set used in this analysis (https://doi.org/10.15468/dl.igi8k0; Global Biodiversity Information Facility, 2019).

## APPENDIX 1 A best practices checklist for developing a phenology scoring rubric for online citizen science photographs.

We list a series of recommendations that form a core set of best practices when developing and implementing a scoring rubric for annotating plant phenology from online citizen science photographs. The list should serve for any online natural history photographic resource but was developed with a focus on iNaturalist.

**Develop a consensus and standards‐based scoring system:**

1. We strongly suggest aligning definitions and terminology to well‐defined standards such as the Plant Phenology Ontology (Stucky et al., 2018).
2. We recommend utilizing scoring protocols, such as those defined by Yost et al. (2018), which were developed for digitized herbarium sheets but apply here as well. As with #1 above, those protocols ensure that scoring uses standard terms.
3. iNaturalist records often can be used to report absence, which is particularly useful for modeling climatic drivers of phenology. Definitive absence requires scoring if a whole plant is visible in the photograph, which we strongly recommend capturing.
4. We advocate a muti‐scorer approach (i.e., where each image is independently scored at least twice), especially in cases where expertise is lower. In cases of conflict, an expert panel can be used to resolve issues.

**Develop iterative training approaches and record flagging mechanisms for volunteers:**

1. We suggest an iterative procedure for testing volunteer's internal definitions. This iterative procedure involves multiple testing rounds and checks for scoring consistency.
2. Consistency checks can reveal photographs that are particularly difficult to score, and discussing those photographs as a group will help refine scoring rubrics.
3. Adding a way to flag challenging records during the actual scoring assures the scorers can bring those records to the group for discussion.

**Develop efficient scoring mechanisms:**

1. Our efficiency increased markedly when we properly used dependency chains when scoring phenology, and we strongly recommend developing these. For example, if a flower was present, we did not need to score whole plant presence traits.
2. The use of custom software for image annotation proved to be a significant time‐saving device, rather than laboriously shifting between an image viewer and spreadsheet. It also decreased errors in transcribing.

**Publish data and metadata in established repositories:**

We encourage immediate publication of validated phenology annotations into repositories, such as the Global Plant Phenology Portal (https://www.plantphenology.org), to assure best‐possible integration and re‐use. More work to set up licensing and rights, along with full metadata about methods, will further improve downstream re‐use.
