# Supplementary material for: Methods for broad‐scale plant phenology assessments using citizen scientists’ photographs
Source: Appl Plant Sci. 2020 Jan 8;8(1):e11315. doi: 10.1002/aps3.11315 (PMC6976896; doi:10.1002/aps3.11315)

APPENDIX S2. Screenshot showing additions to the Global Plant Phenology Data Portal based on our work. Phenology annotations created by this project are discoverable by choosing the "Image Scoring Records from iNaturalist" source and searching for the genus *Yucca*. The portal returns a map interface by default, but records can be viewed in table-mode and downloaded.

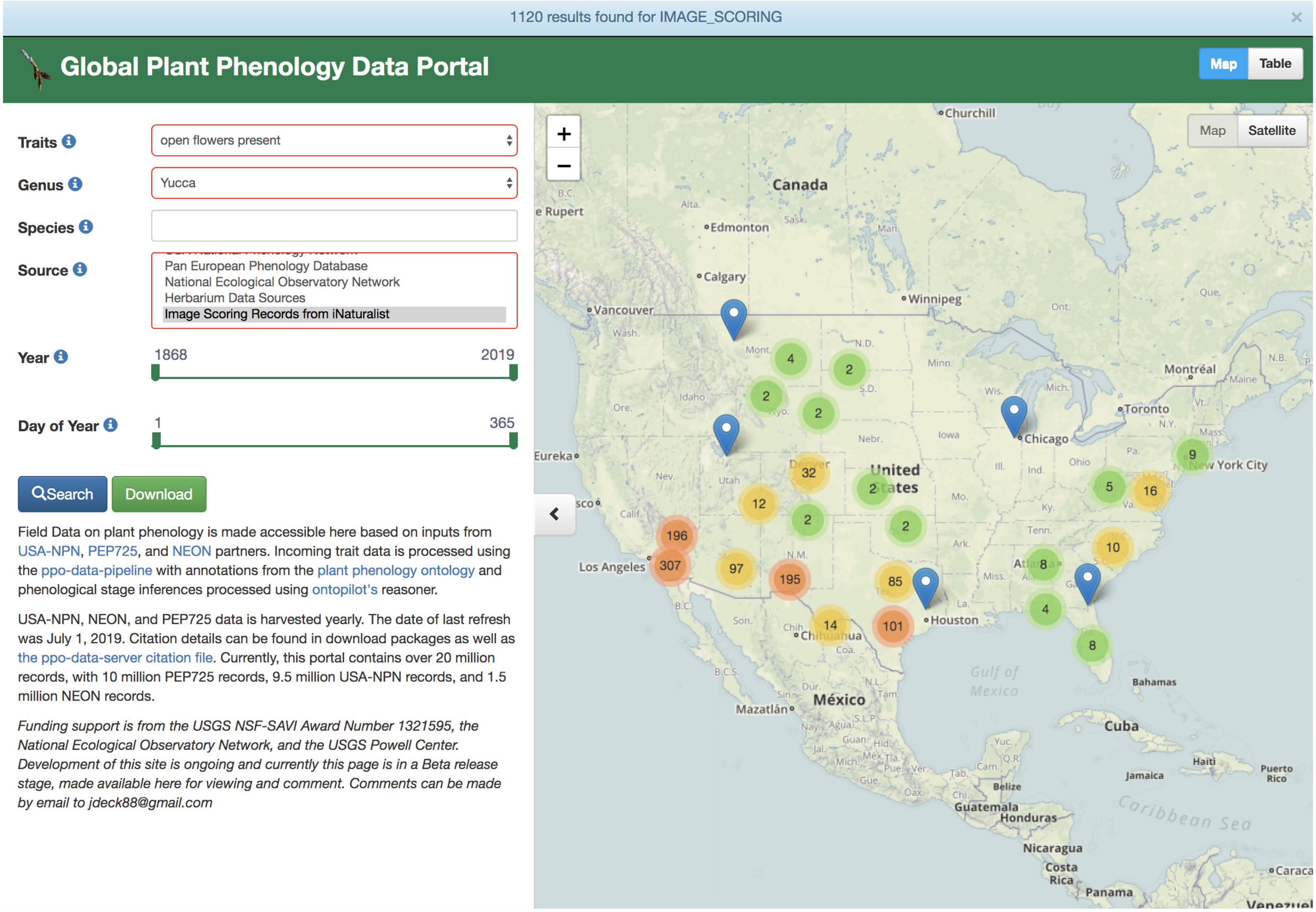

Supplement: Supplementary file 2 — APPENDIX S2. A screenshot showing additions to the Global Plant Phenology Data Portal based on our work. Phenology annotations created by this project are discoverable by choosing the “Image Scoring Records from iNaturalist” source and searching for the genus Yucca. The portal returns a map interface by default, but records can be viewed in table‐mode and downloaded. [file APS3-8-e11315-s002.pdf]
